# Integrating morphometrics and seminal plasma metabolomics to predict fertility in Yoruba Ecotype × Sussex crossbred cocks

**DOI:** 10.14202/vetworld.2025.2699-2711

**Published:** 2025-09-11

**Authors:** Adeyinka Oye Akintunde, Stacey Ogheneovo Ohwofa, Imam Mustofa, Lois Chidinma Ndubuisi-Ogbonna, Samson Oluwole Oyewumi, Aswin Rafif Khairullah

**Affiliations:** 1 Department of Agriculture and Industrial Technology, Babcock University, Ilishan-Remo, Ogun State, Nigeria; 2 Division of Veterinary Reproduction, Faculty of Veterinary Medicine, Universitas Airlangga, Kampus C Unair, Mulyorejo, Surabaya 60115, Indonesia; 3 Research Center for Veterinary Science, National Research and Innovation Agency (BRIN), Jl. Raya Bogor Km. 46 Cibinong, Bogor 16911, West Java, Indonesia

**Keywords:** metabolomics, morphometrics, sperm quality, Sussex crossbreed, food security, Yoruba ecotype

## Abstract

**Background and Aim:**

Enhancing poultry reproductive performance is essential for improving productivity and addressing protein shortages in developing regions. Yoruba ecotype chickens (YECs) are resilient but limited in commercial potential due to small body size and low egg yield. This study assessed the fertilizing potential of YEC × Sussex (SS) crossbred cocks by integrating conventional reproductive morphometrics with seminal plasma metabolite profiling to identify potential biomarkers of sperm quality.

**Materials and Methods:**

Thirty 24-week-old YEC × SS cocks (2842.86 ± 137.33 g) were evaluated over 28 days. Semen was collected through abdominal massage, and semen volume, sperm concentration, and testosterone levels (enzyme-linked immunosorbent assay) were recorded. Testicular and epididymal morphometrics, densities, and sperm reserves were measured. Seminal plasma metabolites were analyzed using high-performance liquid chromatography-mass spectrometry. Pearson correlations and linear regression models were applied to predict testosterone concentration from semen and morphometric traits.

**Results:**

The left testis was heavier (8.00 g) and larger in volume (12.77 mL) than the right (6.75 g; 1.35 mL). Spermatozoa reserves averaged 0.20 × 10^9^, with a strong positive correlation with testis volume (r = 0.998, p < 0.01) and a moderate negative correlation with daily sperm production (r = –0.585, p < 0.01). Testosterone concentration prediction from live weight, semen volume, and sperm concentration achieved high accuracy (R^2^ = 0.829). Thirteen seminal plasma metabolites were identified, including ascorbic acid, quercetin, epicatechin, citric acid, and procyanidin B2 – compounds linked to antioxidant defense, energy metabolism, and sperm viability.

**Conclusion:**

YEC × SS crossbred cocks exhibit favorable reproductive morphometrics, strong correlations between testis volume and sperm reserves, and a metabolite profile enriched in fertility-enhancing antioxidants. Predictive models using basic semen traits can reliably estimate testosterone levels, while identified metabolites have potential as biochemical markers for breeding selection. Integrating morphometric and metabolomic profiling can refine breeding strategies, improve artificial insemination outcomes, and enhance the genetic improvement of local poultry breeds.

## INTRODUCTION

Poultry and its products, such as eggs, are among the most affordable and valuable sources of animal protein recommended for human consumption. Local Nigerian chicken breeds can be used to develop strains suitable for tropical environments [1]. Numerous efforts have been made to crossbreed local chickens with exotic breeds. Local Nigerian breeds, particularly the Yoruba ecotype chicken (YEC), have been investigated for their adaptability to tropical climates, leading to crossbreeding attempts with exotic lines.

Ademola *et al*. [2] evaluated the survivability of YEC, Sussex (SS), Goliath (GG), Marshall, and their crossbreed progeny in Nigeria under humid tropical conditions. They concluded that YEC exhibited greater resilience during the early growth stages, whereas GG crossbreeding improved performance in the later growth phases. Furthermore, Ademola *et al*. [3] examined the egg production and quality traits of Yoruba, SS, and GG chickens, along with their crossbred offspring, under humid tropical conditions. The study found that crossbreeding improved the egg production of YECs, suggesting that YEC × SS crossbreds could be advantageous for egg production in Nigeria. Compared with exotic breeds, these crossbreds are more resilient, require less medical attention, and exhibit greater resistance to ectoparasites and protozoa, with a strong ability to adapt to harsh environmental conditions.

Ademola *et al*. [4] highlighted variations in egg quality due to crossbreeding and emphasized the resilience of native crossbreds, recommending the evaluation of their sperm characteristics given the increasing role of artificial insemination (AI) in poultry breeding. AI, which enables the broader use of genetically superior, high-performing cockerels [5], was the first biotechnological tool used to enhance poultry productivity. In 1899, Ivanov initiated the development of AI as a practical method for domestic animals, including poultry, in Russia [5]. Ivanov successfully produced fertile chicken eggs using semen extracted from the ductus deferens of a cockerel, marking the first application of AI in birds over a century ago. This innovation facilitated semen collection and application in poultry reproduction. AI is vital to the poultry industry because it enhances productivity, supports food security, and reduces the cost and inefficiencies associated with maintaining large numbers of males in rural poultry systems. As an efficient mating system, AI is widely recommended by Bekele *et al*. [6]. In poultry farming, AI is considered a key technology because it enables the efficient use of males, which is often limited in natural mating systems [7].

Inadequate animal protein intake remains a significant nutritional challenge in Nigeria and other developing nations. YEC thrive in tropical environments because of their adaptability, fertility, and resilience [3]. However, their small body size, low egg weight, and slow growth rate limit their commercial potential in Nigeria. Despite their hardiness and adaptability, the limited genetic potential of YECs remains a major constraint to their commercial use [8]. Nevertheless, the hardiness of YEC makes them suitable for rearing in Nigeria, thereby necessitating genetic improvement through selective breeding. Crossbreeding leverages the adaptive traits of indigenous animals. YEC × SS crossbreds are considered highly promising for improving local chicken breeds, as recommended by Ademola *et al*. [3], but there is no study that has elucidated the sperm viability potentials of YEC × SS crossbred cocks. Evaluating the sperm quality of YEC × SS crossbreds is essential for determining their male reproductive potential, which is an important component of breeding assessments.

Although YEC × SS crossbreds have shown superior adaptability and productive performance, there is currently no published research detailing their sperm quality, reproductive morphometry, and seminal plasma biochemical composition. Existing studies on Nigerian indigenous and crossbred chickens have largely focused on survivability, growth, and egg production traits [2–4], leaving reproductive physiology, particularly functional sperm competence, underexplored.

Traditional semen evaluation, which relies on motility, morphology, concentration, and viability, has limitations, especially in avian species where sperm are small and elongated, making morphology assessments difficult. These conventional parameters also do not always correlate strongly with actual fertility outcomes. There is a critical gap in applying seminal plasma metabolomics to indigenous chicken crossbreeds, despite its potential to identify biomarkers linked to energy metabolism, oxidative stress resistance, membrane fluidity, and capacitation-like changes. Such profiling could bridge the gap between sperm structural appearance and actual fertilizing ability, thereby refining breeding selection strategies.

This study aimed to assess the fertilizing potential of YEC × SS crossbred cocks by integrating traditional reproductive morphometrics with seminal plasma metabolite profiling. Specifically, the objectives were to:

1. Characterize testicular and epididymal morphometrics, semen traits, and testosterone levels in YEC × SS crossbred cocks
2. Evaluate correlations between morphometric traits, semen quality parameters, and hormone concentrations
3. Identify and annotate seminal plasma metabolites with potential biomarker value for male fertility
4. Develop predictive models linking conventional semen traits to testosterone concentration for use in breeding selection.

To the best of our knowledge, this is one of the first studies to integrate reproductive morphometry, hormone prediction, and metabolomics in YEC × SS crossbreds, offering a more precise approach to poultry fertility assessment and selection.

## MATERIALS AND METHODS

### Ethical approval

All procedures were approved by the Ethics Commission of Airlangga University (Certificate No. 0574/HRECC.FDOM/V/2024).

### Study period and location

This study was conducted from June 2024 to February 2025 at the Teaching and Research Farm Poultry Unit, Babcock University, Ilishan-Remo, Ogun State, Nigeria. The farm is located in the southwestern rainforest belt of Nigeria, with an annual rainfall of 1,500 mm, an altitude of about 300 mm above sea level, and an annual mean temperature of 27°C. Geographically, it lies at longitude 3°43’12’’E of the Greenwich meridian and latitude 6°53’23’’N of the equator.

### Animal housing and management

The poultry pen was cleaned, disinfected, and left to dry for 2 weeks before the arrival of the birds. The birds were acclimatized for a period of 14 days. Drinkers and feeders were also thoroughly cleaned and disinfected. Feed and water were provided *ad libitum* throughout the study period.

### Experimental animals and sampling procedures

Crossbreeding between male YEC and female SS had previously been carried out, and the resulting F1 offspring formed the study population. Thirty 24-week-old YEC × SS crossbred cocks were randomly selected from a total population of 96 cocks.

### Semen collection and analysis

Semen was collected twice weekly between 06:00 and 08:00 h using the abdominal massage technique, which induces cloacal eversion. Ejaculates were collected in graduated tubes, and data were recorded individually for each bird before averaging.

- Semen volume was measured directly using Pyrex graduated collection tubes (IMV Technologies Group, France).
- Sperm concentration was determined by diluting semen samples with sodium citrate, loading them onto a hemocytometer, and observing at 400× magnification under a microscope.

### Hormonal assay

Blood samples were collected from the jugular vein into heparinized tubes, centrifuged at 1,000 × *g* for 15 min, and serum was separated and stored at −4°C until analysis. Testosterone concentration was determined by enzyme-linked immunosorbent assay using a commercial kit (ELISA, AccuBind ELISA Microwells Testosterone test system with the product code: 3725-300, Monobind Inc., Lake Forest, CA, USA). The sensitivity of the hormone detected per assay tube was 0.05 ng/mL.

### Testicular and epididymal morphometric analysis

Testes and epididymides were carefully excised and weighed using an analytical balance. Measurements included:

- Length: Measured with a thread aligned to a calibrated ruler.
- Volume: Metabolomics, morphometrics, sperm quality, SS crossbreed, testosterone prediction, Yoruba ecotype determined by water displacement in a graduated cylinder based on Archimedes’ principle [9].
- Density: Calculated as organ weight divided by volume.
- Diameter and circumference: Measured using a flexible measuring tape.

Relative organ weights (left, right, and paired) were calculated as:


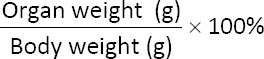


Sperm reserves were estimated by homogenizing each testis and epididymis in 1 mL of 0.154 M NaCl, filtering through gauze, and diluting to 1:30 with normal saline before hemocytometer counting at 400× magnification.

Daily sperm production (DSP) was calculated as:


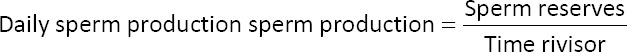


Potential fertilizing capacity per ejaculate was estimated using the formula from Akintunde *et al*. [10], based on motile sperm count per standard insemination dose.

### Metabolite profiling

Seminal plasma samples were analyzed using a Shimadzu high-performance liquid chromatography system with an SPD-M10AVP diode array detector (Shimadzu Corporation, Kyoto, Japan). Analytical conditions were:

- Column: Reverse-phase C-18 (Phenomenex Luna, 150 × 4.6 mm, 5 μm)
- Flow rate: 1 mL/min
- Injection volume: 20 μL
- Detection wavelength: 660 nm
- Mobile phase: Acetonitrile/water (54:46).

### Statistical analysis

Least squares means and standard error values for each parameter were calculated using Microsoft Excel 2007 (Microsoft Corp., San Jose, CA, USA). Pearson correlations between testicular morphometrics and semen quality traits were analyzed using the Statistical Package for the Social Sciences (SPSS) version 20 (IBM Corp., NY, USA), with significance set at p < 0.05.

Regression models for predicting sperm parameters from morphometric traits were developed using forward stepwise regression [11]:

Y = a + b_1_X_1_ +… + b_k_X_k_

Where X_i_ represents the predictor variable, b_k_ the partial regression coefficient, the intercept, and Y the dependent variable.

Data were processed using Class-VP software version 6.10 (Shimadzu Corporation, Japan). Mass spectrometric analysis was performed with a Bruker Esquire 2000 Plus (Bruker Daltonics, Massachusetts, USA) in positive electrospray ionization mode (capillary voltage: 4.5 kV; skimmer voltage: 40 eV). This analysis provided comprehensive information on testicular and epididymal parameters, sperm reserves, and potential biomarkers for reproductive assessment in YEC × SS crossbred cocks.

## RESULTS

### Sperm characteristics of YEC × SS crossbred cocks

Table 1 provides a comprehensive summary of sperm characteristics in YEC × SS cocks, including organ weights, volumes, dimensions, densities, and other reproductive parameters. Values are expressed as means with standard deviations, reflecting variability within the sample population.

**Table 1:** Sperm characteristics of YEC × SS cocks.

Parameter	YEC
Live weight (g)	2842.86 ± 137.33
Right testis weight (g)	6.75 ± 0.48
Left testis weight (g)	8.00 ± 1.00
Paired testis weight (g)	14.80 ± 1.31
Right epididymis weight (g)	0.37 ± 0.05
Left epididymis weight (g)	0.27 ± 0.02
Paired epididymis weight (g)	0.63 ± 0.07
Right testis volume (mL)	1.35 ± 2.50
Left testis volume (mL)	12.77 ± 1.30
Right epididymal volume (mL)	0.13 ± 0.02
Left epididymal volume (mL)	0.10 ± 0.00
Paired epididymal volume (mL)	0.22 ± 0.02
Right testis length (cm)	4.50 ± 0.58
Left testis length (cm)	4.50 ± 0.29
Right testis circumference (cm)	5.80 ± 0.28
Left testis circumference (cm)	5.80 ± 0.47
Right testis diameter (cm)	1.85 ± 0.09
Left testis diameter (cm)	1.85 ± 0.15
Right testis density (g/mL)	0.56 ± 0.11
Left testis density (g/mL)	0.60 ± 0.07
Paired testis density (g/mL)	0.59 ± 0.09
Right epididymal density (g/mL)	3.08 ± 0.39
Left epididymal density (g/mL)	2.70 ± 0.22
Paired epididymal density (g/mL)	2.86 ± 0.29
Spermatozoa reserves (g)	0.20 ± 0.01
Relative testis weight (%)	0.57 ± 0.06
Relative epididymal weight (%)	0.02 ± 0.00

The values are presented as group mean and standard error of sample. YEC = Yoruba ecotype chickens, SS = Sussex.

The average live weight was 2,842.86 g, indicating a robust body size for this crossbreed. The left testis was generally heavier (8.00 g) and had a larger volume (12.77 mL) compared to the right testis (6.75 g and 1.35 mL, respectively). The paired testis weight was 14.75 g, representing the combined mass of both testes.

The right epididymis weighed more (0.37 g) than the left (0.26 g), with a total paired weight of 0.63 g. Similarly, the right epididymal volume (0.12 mL) exceeded the left (0.10 mL), resulting in a paired volume of 0.22 mL.

Epididymides had higher densities (3.07 g/mL right; 2.65 g/mL left) than testes (0.55 g/mL right; 0.63 g/mL left), suggesting a compact structure and a functional role in sperm maturation and storage. Spermatozoa reserves measured 0.20 g, providing an estimate of reproductive potential and sperm production efficiency. The relative testis weight was 0.57%, whereas the relative epididymal weight was 0.02%, indicating the proportion of body weight dedicated to reproductive organs.

### Correlations between morphometric and reproductive traits

Table 2 shows:

**Table 2:** Relationships of spermiogramic parameters of YEC × SS crossbred cocks.

Correlation Coefficients	Live weight	Testicular weight	Epididymal weight	Testis volume	Epididymal volume	Testicular density	Epididymal density	Spermatozoa reserves-testis	Spermatozoa reserves: Epididymal	Daily sperm production (Testis)	Daily sperm production—epididymal
Live weight	1.00										
Testicular weight	−0.27	1.00									
Epididymal weight	0.22	−0.77	1.00								
Testis volume	−0.83	0.50	0.70	1.00							
Epididymal volume	0.22	−0.95*	0.54	−0.31	1.00						
Testis density	0.63	0.21	0.27	−0.73	−0.43	1.00					
Epididymal density	−0.01	0.19	0.48	0.41	−0.48	0.73	1.00				
Spermatozoa reserves: Testis	0.86	0.26	0.15	−0.59	−0.30	0.78	0.15	1.00			
Spermatozoa reserves: Epididymal	−0.81	0.48	−0.71	0.99**	−0.27	−0.75	−0.46	−0.58	1.00		
Daily sperm production (testis)	0.86	0.26	−0.15	−0.59	−0.30	0.78	0.15	1.00	−0.58	1.00	
Daily sperm production—Epididymal	−0.81	0.48	−0.71	0.99**	−0.27	0.75	−0.46	−0.58	1.00**	−0.58	1.00

** p < 0.01. YEC = Yoruba ecotype chickens, SS = Sussex.

- A positive correlation between live weight and epididymal weight (r = 0.220)
- A strong negative correlation between live weight and testis volume (r = 0.828)
- A very strong positive correlation between testis volume and spermatozoa reserves (r = 0.998, p < 0.01)
- A moderate negative correlation between spermatozoa reserves and DSP (r = 0.585).

Table 3 shows positive correlations between testosterone concentration and:

- Testis weight (r = 0.242)
- Spermatozoa reserves (r = 0.657)
- Testis volume (r = 0.727)
- Live weight (r = 0.809).

**Table 3:** Correlation coefficient of sperm parameters with YEC × SS crossbred cocks.

Parameter	Testosterone	Testis weight	Spermatozoa reserves (PT)	Testis volume	Live weight
Testosterone	1.00				
Testis weight	0.24	1.00			
Spermatozoa reserves (PT)	0.66	0.15	1.00		
Testis volume	0.73	0.50	0.62	1.00	
Live weight	0.81	0.11	0.60	0.54	1.00

The values are presented as correlation coefficient. YEC = Yoruba ecotype chickens, SS = Sussex.

A positive correlation was also observed between testis weight and spermatozoa reserves (r = 0.154).

### Regression models for predicting testosterone concentration

Table 4 presents regression equations linking live weight, semen volume, and sperm concentration to testosterone levels. The R^2^ value of 0.829 indicates high predictive accuracy for testosterone concentration using these variables.

**Table 4:** Prediction of testosterone concentration using vital semen parameters of YEC × SS crossbred cocks.

Independent variable	Dependent variable	Regression equation	R^2^	SE
Live weight	Testosterone	8.11 + 0.32LW + 0.37SV + 0.03SC	0.83	0.47
Semen volume				
Sperm concentration				

LW = Live weight, SV = Semen volume, SC = Transpire concentration, R2 = Variance explained, SE = Associated standard error, YEC = Yoruba ecotype chickens, SS = Sussex.

Table 5 presents regression equations predicting testosterone concentration from testis volume and spermatozoa reserves, with an R^2^ value of 0.673, suggesting a moderately strong predictive relationship between spermogram parameters and testosterone levels.

**Table 5:** Prediction of testosterone concentration using vital semen parameters of YEC × SS crossbred cocks.

Independent variable	Dependent variable	Regression equation	R^2^	SE
Semen volume	Testosterone	2.98 + 0.45SV + (−12.58SC)	0.67	0.00
Sperm concentration				

SV = Semen volume, SC = Sperm concentration, R^2^ = Variance explained, SE = Associated standard error, YEC = Yoruba ecotype chickens, SS = Sussex.

### Seminal plasma metabolite profile

Table 6 lists the metabolites detected in YEC × SS cocks, including RT, molecular formulas, molecular weights, peak areas, compound weight percentages, and mass-to-charge ratios (m/z). These metabolites are relevant to both general physiological health and reproductive performance.

**Table 6:** Metabolites in the seminal plasma of YEC × SS crossbred cocks.

Peak	RT (min)	Compound detected	Mol. Formula	MW	Peak area (%)	Comp %wt	m/z	Structure
1	1.29	3-Hydroxybenzoic acid	C_7_H_6_O_3_	138.00	2.65 ± 0.01	2.70 ± 0.01	65.00, 93.00, and 138.00	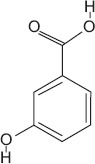
2	8.42	Benzoic acid, 3 ,4- dihydroxy- protocatechuic acid	C_7_H_6_O_4_	154.00	8.49 ± 0.76	3.28 ± 0.42	77.00, 87.00, 154.00	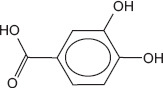
3	8.72	Citric acid	C_6_H_8_O_7_	192.00	11.93 ± 3.76	10.29 ± 0.13	43.00, 129.00, and 192.00	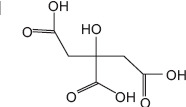
4	9.25	Gallic acid	C_7_H_6_O_5_	170.00	7.03 ± 5.04	5.38 ± 2.38	57.00, 126.00, 170.00	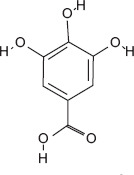
5	12.42	Malic acid	C_4_H_6_O_5_	134.00	5.46 ± 2.10	5.66 ± 5.49	43.00, 71.00, 134.00	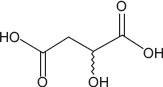
6	13.18	(+)-catechin	C_15_H_14_O_6_	290.00	7.71 ± 5.01	3.36 ± 2.45	139.00, 152.00, 290.00	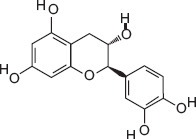
7	14.51	(−)-Epicatechin	C_15_H_14_O_6_	290.00	13.83 ± 3.85	5.77 ± 2.28	147.00, 179.00, 290.00	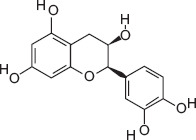
8	17.01	Quercetin	C_15_H_10_O_7_	302.00	8.79 ± 1.62	9.67 ± 2.34	77.00, 133.00, 302.00	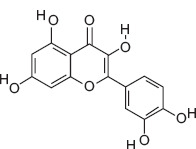
9	18.83	Myricetin	C_15_H_10_O_8_	318.00	5.298 ± 0.50	11.04 ± 1.10	77.00, 133.00, 318.00	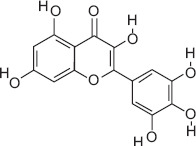
10	12.98	Chlorogenic acid	C_16_H_18_O_9_	327.00	5.10 ± 0.65	16.93 ± 0.10	147.00, 245.00, 327.00	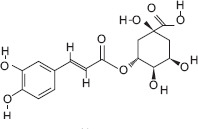
11	12.99	Procyanidin B2	C_30_H_26_O_12_	578.00	17.32 ± 2.02	9.22 ± 4.69	127.00, 411.00, 578.00	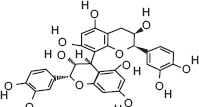
12	9.25	Luteolin	C_15_H_10_O_6_	286.00	6.25 ± 0.05	11.21 ± 1.10	65.00, 153.00, 286.00	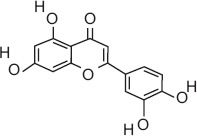
13	17.01	Ascorbic acid	C_6_H_8_O_6_	176.00	12.50 ± 0.91	8.13 ± 1.22	43.00, 141.00, and 176.00	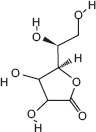

RT = Reaction time, Comp = Compound, MW = Molecular weight, m/z = Mass to charge ratio, YEC = Yoruba ecotype chickens, SS = Sussex.

### Functional significance of major metabolites

- Ascorbic acid (Vitamin C) - A potent antioxidant that protects sperm DNA from oxidative damage, improves sperm motility, and enhances fertilization success.
- Quercetin - Flavonoid with antioxidant and anti-inflammatory effects; improves sperm count, motility, and morphology.
- (−)-Epicatechin and (+)-Catechin - Reduce lipid peroxidation in sperm membranes, maintain membrane integrity, and support capacitation.
- Procyanidin B2 - Polyphenolic compound linked to improved sperm motility and membrane stability under oxidative stress.
- Citric acid - Maintains semen pH and osmolarity; serves as an energy substrate for sperm metabolism.
- Gallic acid - Protects reproductive tissues from oxidative injury and supports sperm viability.
- 3-Hydroxybenzoic acid and protocatechuic acid - Scavenge reactive oxygen species, reducing oxidative stress in spermatozoa.
- Malic acid - Participates in the Krebs cycle, providing energy for sperm motility.
- Myricetin, Luteolin, and Chlorogenic acid - Natural antioxidants that enhance sperm membrane stability and reduce oxidative damage during storage or transport.

The presence of these bioactive compounds in seminal plasma suggests a strong antioxidant and metabolic support system in YEC × SS cocks, potentially contributing to their reproductive resilience and fertilizing capacity. These metabolites may serve as biomarkers for selecting elite breeding males in AI programs.

## DISCUSSION

### Testis asymmetry in YEC × SS cocks

The presented data provide a detailed overview of the reproductive anatomy and fertilizing potential of YEC × SS cocks. Asymmetries in testicular and epididymal measurements, including differences in density and weight, form the basis for understanding correlations with fertility and reproductive success. Such parameters are valuable for developing selective breeding strategies aimed at improving reproductive traits and genetic quality in poultry.

The mean live weight of YEC × SS cocks was 2,842.86 ± 137.33 g, which serves as a reference for evaluating bird health and size, factors important in breeding and production studies [12]. Live weight can influence reproductive traits and is an indicator of general well-being and nutritional adequacy.

### Morphometric differences between left and right reproductive organs

A notable difference was observed in testis weights: The left testis (8.00 ± 1.00 g) was heavier than the right (6.75 ± 0.48 g), with a paired weight of 14.75 ± 1.31 g. Volume asymmetry was also evident, with the left testis (12.77 ± 1.30 mL) larger than the right (1.35 ± 2.50 mL). Such asymmetry is common in avian species and may reflect functional specialization, where one testis, often the left, is more active in spermatogenesis [13].

For the epididymides, the right weighed more (0.37 ± 0.05 g) than the left (0.26 ± 0.02 g), with a paired weight of 0.63 ± 0.06 g. Volume followed the same trend: Right (0.12 ± 0.02 mL) greater than left (0.10 ± 0.00 mL). These differences are important because the epididymis plays a key role in sperm maturation and storage [14].

### Measurements of size and density

Testis length was identical on both sides (4.50 ± 0.58 cm right; 4.50 ± 0.29 cm left), with similar circumferences (5.80 ± 0.27 cm right; 5.80 ± 0.46 cm left) and diameters (1.85 ± 0.09 cm right; 1.85 ± 0.15 cm left).

Density differed between organs: The right testis (0.55 ± 0.11 g/mL) was less dense than the left (0.63 ± 0.06 g/mL), while the right epididymis (3.07 ± 0.38 g/mL) was denser than the left (2.65 ± 0.22 g/mL), indicating potential differences in tissue compactness and spermatogenic activity [15].

### Spermatozoa reserves and relative organ weights

Spermatozoa reserves averaged 0.20 ± 0.01 g, reflecting the stored sperm quantity in the epididymis, a critical determinant of fertilizing capacity. Relative testis weight (0.57% ± 0.05%) and epididymal weight (0.02% ± 0.00%) indicate the proportion of body weight invested in reproductive function [16].

Table 1 data highlight these asymmetries, which are common in birds and useful for breeding evaluations. Testis size remains a primary indicator of reproductive capacity and spermatogenic activity [17].

### Relationship with testosterone and fertility indicators

This study demonstrated that testosterone concentration could be predicted from live weight, semen volume, and sperm concentration, aligning with known patterns of testicular asymmetry in birds [18]. Androgen levels, especially testosterone, influence testis weight and secondary sexual traits [19, 20].

Banakar *et al*. [19] reported no direct link between testosterone and fertility but noted testosterone decline after 30 weeks of age. Escorcia *et al*. [16] observed marked testosterone drops by 40 weeks in broiler breeders, with corresponding reductions in testis weight. These findings are consistent with the findings of Sun *et al*. [20], who also linked testosterone levels to testis size but not sperm motility.

### Comparative morphometry with previous studies

The testis weights recorded here exceeded those reported by Akintunde *et al*. [10] for YEC, where the left was 3.41 ± 0.12 g, the right 1.96 ± 0.10 g, and the paired 5.37 ± 0.16 g. Although the left epididymis weight here was lower than 0.33 ± 0.04 g reported for YEC, right and paired weights were higher.

Left testis volume (12.77 mL) exceeded the 3.69 ± 0.12 mL reported for YEC, whereas the right (1.35 mL) was lower than the 2.74 ± 0.14 mL reported for YEC. Relative testis weight (0.57%) was higher than 0.45% ± 0.03% for YEC, while relative epididymal weight (0.02%) was lower than 0.04% ± 0.00% [10].

Compared with Adamu *et al*. [21], who reported 11.74 ± 4.53 mL for local Sahel cocks, the present values vary, likely due to environmental factors.

### Importance of morphometric traits

Akintunde *et al*. [22] and Janosikova *et al*. [14] highlighted morphometric traits as strong indicators for breeding potential. Montes-Garrido *et al*. [9] found testicular dimensions useful for predicting sperm output. Banakar *et al*. [19] proposed functional thresholds: <5 g (nonfunctional), 6–10 g (borderline), >10 g (functional).

In this study, YEC × SS testes fall in the functional range. There are positive correlations between testis and live weight mirror findings from Escorcia *et al*. [16], Ahemen *et al*. [23], and Talebi *et al*. [24].

### Testis volume and sperm output

Testis volume correlates positively with spermatozoa reserves because larger testes contain more seminiferous tubules for spermatogenesis [9, 25]. This measure is a reliable predictor of sperm output and the ability to sustain frequent inseminations [26].

### Seminal plasma metabolites and fertility significance

The identified metabolites in YEC × SS cocks are rich in antioxidants and bioactive compounds that support sperm health and fertility. These include:

- 3-hydroxybenzoic acid - Antioxidant reducing oxidative stress [27, 28]
- Protocatechuic acid - Anti-inflammatory and antioxidant, improves motility and viability [29]
- Citric acid - Maintains semen pH/osmolarity, energy substrate for sperm [30, 31]
- Gallic acid - Protects reproductive tissues from oxidative damage [32–35]
- Malic acid - Supports energy production through the Krebs cycle [36]
- Catechin/epicatechin - Preserve membrane integrity, reduce lipid peroxidation [37]
- Quercetin - Improves sperm quality, reduces oxidative stress [38, 39]
- Myricetin - Antioxidant protecting spermatozoa [40]
- Chlorogenic acid - Antioxidant and anti-inflammatory [41]
- Procyanidin B2 - Enhances motility and membrane stability [42]
- Luteolin - Anti-inflammatory, supports reproductive health [43, 44]
- Ascorbic acid (Vitamin C) - Reduces DNA damage, improves motility [28, 45].

### Breeding implications

The presence of ascorbic acid, quercetin, epicatechin, citric acid, and procyanidin B2 in seminal plasma suggests a strong antioxidant defense and metabolic support system. Higher concentrations of these compounds may indicate genetic lines with enhanced sperm resilience and fertilizing capacity, especially under oxidative stress, a common challenge in intensive poultry systems [46, 47].

These metabolites may serve as biochemical biomarkers for elite male selection, enabling integration of seminal plasma metabolomics with conventional selection indices to improve reproductive outcomes and genetic gain in poultry breeding programs.

## CONCLUSION

This study demonstrated that Yoruba Ecotype × SS (YEC × SS) crossbred cocks possess favorable reproductive morphometrics, with the left testis consistently larger and heavier than the right, indicating functional asymmetry common in avian species. Strong positive correlations were found between testis volume and spermatozoa reserves (r = 0.998, p < 0.01) and between testosterone concentration and morphometric parameters such as live weight, testis volume, and sperm reserves. Regression models showed high predictive accuracy (R^2^ = 0.829) for estimating testosterone levels from live weight, semen volume, and sperm concentration. Seminal plasma profiling identified 13 bioactive metabolites, including ascorbic acid, quercetin, catechins, citric acid, and procyanidin B2, linked to antioxidant defense, energy metabolism, and sperm viability.

The integration of reproductive morphometrics with seminal plasma metabolomics offers a reliable dual-parameter framework for breeding selection. This approach enables poultry breeders to identify elite males with superior fertility potential, optimize AI programs, and reduce dependence on exotic breeds while improving productivity in local poultry systems.

The study’s strengths include being the first comprehensive evaluation combining morphometric, hormonal, and metabolomic profiles in YEC × SS cocks, identification of specific metabolites with biomarker potential for fertility selection, and the development of predictive models with high statistical correlation. However, the research is limited by a relatively small sample size, a single study location, and the absence of direct mating or AI trials to confirm fertility outcomes.

Future research should validate the identified metabolites as fertility biomarkers in larger, multi-location trials, assess seasonal and nutritional influences on morphometric and metabolomic profiles, and conduct direct fertility trials to link metabolite patterns with hatchability outcomes. Integrating genomic and proteomic approaches could further enhance multi-omics-based breeding selection strategies.

YEC × SS crossbred cocks show strong potential as a genetic resource for enhancing reproductive efficiency in Nigerian poultry systems. By combining traditional reproductive assessments with advanced metabolomic profiling, this study provides a scientifically grounded framework for targeted male selection, contributing to improved productivity, genetic gain, and food security in tropical poultry production.

## AUTHORS’ CONTRIBUTIONS

AOA and SOO: Management of experimental animals and data collection, management, and analysis. AOA, ARK, and IM: Conceptualized and designed the study, data analysis, and drafted the manuscript. LCNO and SOOy: Visualized and revised the manuscript. All authors have read and approved the final manuscript.

## References

[1] Manyelo T.G, Selaledi L, Hassan Z.M, Mabelebele M (2020). Local chicken breeds of Africa:Their description, uses and conservation methods. Animals.

[2] Ademola A.A, Fayeye T.R, Akintunde A.O, Chiemezie V.O, Jubril A.E (2020). Survivability of pure and cross bred chickens at early and late growth phases in Nigeria humid tropics. Niger. J. Anim. Prod.

[3] Ademola A.A, Fayeye T.R, Akintunde A.O, Chimezie V (2023). Egg production and egg quality characteristics of Yoruba, Sussex, and Goliath chickens and their crossbred progenies under humid tropical climate. Ovozoa J. Anim. Reprod.

[4] Ademola A.A, Fayeye T.R, Akintunde A.O, Chimezie V (2024). Principal component analysis of egg parameters in Yoruba Ecotype, Sussex and their cross bred chickens. Res. Bio.

[5] Mohan J, Sharma S.K, Kolluri G, Dhama K (2018). History of artificial insemination in poultry, its components and significance. World's Poult. Sci. J.

[6] Bekele B, Melesse A, Esatu W, Dessie T ((2022)). Multivariate analysis of morphometric traits to differentiate the indigenous chicken reared under different agroecologies of Ethiopia. Vet. Integr. Sci.

[7] Getachew T (2016). A review article of artificial insemination in poultry. World Vet. J.

[8] Akintunde A.O, Toye A.A (2023). Comparative study on egg characteristics of Yoruba ecotype Nigerian local chickens and isa brown chickens fed graded levels of *Moringa oleifera* seed meal. Agric. Sci. Dig.

[9] Montes-Garrido R, Anel-Lopez L, Riesco M.F, Neila-Montero M, Palacin-Martinez C, Soriano-Úbeda C, Boixo J.C, De Paz P, Anel L, Alvarez M (2023). Does size matter?Testicular volume and its predictive ability of sperm production in rams. Animals (Basel).

[10] Akintunde A.O, Toye A.A, Ademola A.A, Chimezie V.O, Ajayi O.A (2020). Sperm characteristics of Nigeria local cocks and exotic strain of cocks fed graded levels of *Moringa oleifera* seed meal. Trop. Anim. Prod. Invest.

[11] Dahloum L.N.M, Halbouche M, Mignon-Grasteau S (2016). Phenotypic characterization of the indigenous chickens (*Gallus gallus*) in the Northwest of Algeria. Arch. Anim. Breed.

[12] England A, Gharib-Naseri K, Kheravii S.K, Wu S.B (2022). Influence of sex and rearing method on performance and flock uniformity in broilers-implications for research settings. Anim. Nutr.

[13] Althnaian T.A (2022). Morphological studies on the testis, epididymis and vas deferens of Al-ahsa native rooster. Braz. J. Poult. Sci.

[14] Janosikova M, Petricakova K, Ptacek M, Savvulidi F.G, Rychtarova J, Fulka J. Jr (2023). New approaches for long-term conservation of rooster spermatozoa. Poult. Sci.

[15] Matsuzaki M, Sasanami T (2022). Sperm motility regulation in male and female bird genital tracts. J. Poult. Sci.

[16] Escorcia M, Sánchez-Godoy F, Ramos-Vidales D, Medina-Campos O.N, Pedraza-Chaverri J ((2020)). Effect of the age and body weight of the broiler breeders'male on the presentation of oxidative stress and its correlation with the quality of testicular parenchyma and physiological antioxidant levels. Vet. Sci.

[17] Rizzi C, Verdiglione R (2015). Testicular growth and comb and wattles development in three Italian chicken genotypes reared under free-range conditions. Ital. J. Anim. Sci.

[18] Abdul-Rahman I.I, Obese F.Y, Robinson J.E (2018). Testis size and asymmetry in the guinea fowl (*Numida meleagris*):A test of the compensation hypothesis. Avian Biol. Res.

[19] Banakar A, Sharafi M, Li G (2024). Relationship between reproductive indicators and sound structure in broiler breeder roosters. Appl. Anim. Behav. Sci.

[20] Sun Y, Xue F, Li Y, Fu L, Bai H, Ma H, Xu S, Chen J (2019). Differences in semen quality, testicular histomorphology, fertility, reproductive hormone levels, and expression of candidate genes according to sperm motility in Beijing-You chickens. Poult. Sci.

[21] Adamu J, Dauda A, Abbaya H.Y (2019). Effect of genotype and seasons on semen characteristics of three indigenous cock types in the semi-arid zone of Nigeria. Int. J. Avian Wildl. Biol.

[22] Akintunde A.O, Toye A.A, Ademola A.A, Chimezie V.O, Ajayi O.A (2021). Genotype-diet effect on comparative semen parameters of chickens fed graded levels of *Moringa oleifera* seed meal. Mal. J. Anim. Sci.

[23] Ahemen T, Abu A.H, Akuba J.O (2016). Effect of *Gmelina arborea* leaf meal on sperm production and sperm reserves in rabbit bucks. Int. J. Livest. Res.

[24] Talebi A, Alimehr M, Alavi M.H, Najafi G, Simaei N (2018). Comparative study of semen traits and histomorphometric features of testes of broiler breeder males with different phenotypic traits. Vet. Res. Forum.

[25] Preston B.T, Stevenson I.R, Lincoln G.A, Monfort S.L, Pilkington J.G, Wilson K (2012). Testes size, testosterone production and reproductive behaviour in a natural mammalian mating system. J. Anim. Ecol.

[26] Huamani M.C, Palomino C.Y.G, Berrocal H.R.O, Arcce I.M.L, Bellido-Quispe D.K, Leiva A.Y.A, Chaves M.S, De Figueirêdo Freitas V.J (2025). Influence of reproductive season and testicular volume on seminal parameters of alpacas (*Vicugna pacos*). Trop. Anim. Health Prod.

[27] Velika B, Kron I (2012). Antioxidant properties of benzoic acid derivatives against superoxide radical. Free Radicals Antioxidants.

[28] Walke G, Gaurkar S.S, Prasad R, Lohakare T, Wanjari M (2023). The impact of oxidative stress on male reproductive function:Exploring the role of antioxidant supplementation. Cureus.

[29] Cadena-Iñiguez J, Santiago-Osorio E, Sánchez-Flores N, Salazar-Aguilar S, Soto-Hernández R.M, Riviello-Flores M.L, Macías-Zaragoza V.M, Aguiñiga-Sánchez I (2024). The cancer-protective potential of protocatechuic acid:A narrative review. Molecules.

[30] Tanni B, Voundi E.V, Omigbodun A, Aimakhu C.O (2022). Seminal fructose and citric acid concentrations relative to sperm parameters among men for fertility evaluation in Yaoundé, Cameroon. Explor. Med.

[31] Mehaisen G.M.K, Elomda A.M, Hamad S.K, Ghaly M.M, Sun Y, Li Y, Zong Y, Chen J, Partyka A, Nazmi A, Abbas A.O, Stino F.K.R (2022). Effect of dimethylacetamide concentration on motility, quality, antioxidant biomarkers, anti-freeze gene expression, and fertilizing ability of frozen/thawed rooster sperm. Animals (Basel).

[32] Mustofa I, Susilowati S, Suprayogi T.W, Akintunde A.O, Oktanella Y, Purwanto D.A (2023). Epigallocatechin-3-gallate chitosan nanoparticles in an extender improve the antioxidant capacity and post-thawed quality of Kacang goat semen. F1000Res.

[33] Altındağ F, Meydan İ (2021). Evaluation of protective effects of gallic acid on cisplatin-induced testicular and epididymal damage. Andrologia.

[34] Alhazmi A.I, El-Refaei M.F, Abdallah E.A (2024). Protective effects of gallic acid against nickel-induced kidney injury:Impact of antioxidants and transcription factor on the incidence of nephrotoxicity. Ren. Fail.

[35] Susilowati S, Mustofa I, Suparyogi T.W, Akintunde A.O, Purwanto D.A, Wurlina W, Utama S, Mulyati S (2024). Adding chitosan nanoparticles of green tea extract in diluent and thawing temperatures ameliorate the post-thawed quality of Boer buck semen. Asian Pac. J. Reprod.

[36] Qiang F (2015). Effect of malate-oligosaccharide solution on antioxidant capacity of endurance athletes. Open Biomed. Eng. J.

[37] Tvrda E, Straka P, Galbavy D, Ivanic P (2019). Epicatechin provides antioxidant protection to bovine spermatozoa subjected to induced oxidative stress. Molecules.

[38] Li Y, Yao J, Han C, Yang J, Chaudhry M.T, Wang S, Liu H, Yin Y (2016). Quercetin, inflammation and immunity. Nutrients.

[39] Saber T.M, Abd El-Aziz R.M, Ali H.A (2016). Quercetin mitigates fenitrothion-induced testicular toxicity in rats. Andrologia.

[40] Oroojan A.A, Ahangarpour A, Paknejad B, Zareian P, Hami Z, Abtahi S.R (2021). Effects of myricitrin and solid lipid nanoparticle-containing myricitrin on reproductive system disorders induced by diabetes in male mouse. World J Mens Health.

[41] Wang Y, Zhang L, Sohail T, Kang Y, Sun X, Li Y (2022). Chlorogenic acid improves quality of chilled ram sperm by mitigating oxidative stress. Animals (Basel).

[42] Dasiman R, Nor N.M, Eshak Z, Mutalip S.S.M, Suwandi N.R, Bidin H (2022). A review of procyanidin:Updates on current bioactivities and potential health benefits. Biointerface Res. Appl. Chem.

[43] Owumi S.E, Ijadele A.O, Arunsi U.O, Odunola O.A (2020). Luteolin abates reproductive toxicity mediated by the oxido-inflammatory response in Doxorubicin-treated rats. Toxicol. Res. Appl.

[44] Tian C, Liu X, Chang Y, Wang R, Lv T, Cui C, Liu M (2021). Investigation of the anti-inflammatory and antioxidant activities of luteolin, kaempferol, apigenin and quercetin. S. Afr. J. Bot.

[45] Hajjar T, Soleymani F, Vatanchian M (2020). Protective effect of vitamin C and zinc as an antioxidant against chemotherapy-induced male reproductive toxicity. J. Med. Life.

[46] Ahmadi S, Bashiri R, Ghadiri-Anari A, Nadjarzadeh A (2016). Antioxidant supplements and semen parameters:An evidence-based review. Int. J. Reprod. Biomed.

[47] Kaltsas A (2023). Oxidative stress and male infertility:The protective role of antioxidants. Medicina (Kaunas).

